# Investigating the mechanism and the effect of aquaporin 5 (AQP5) on the self-renewal capacity of gastric cancer stem cells

**DOI:** 10.7150/jca.92745

**Published:** 2024-06-11

**Authors:** Peiyao Gao, Amin Chen, Hengjin Tian, Feifan Wang, Na Wang, Kunpeng Ge, Chaoqun Lian, Fengchao Wang, Qiang Zhang

**Affiliations:** 1 Department of Clinical Laboratory, The First Affiliated Hospital of Bengbu Medical University, Bengbu 233004, China.; 2 Key Laboratory of Cancer Research and Clinical Laboratory Diagnosis, Bengbu Medical University, Bengbu 233030, China.; 3 Department of Blood Transfusion, The First Affiliated Hospital of Naval Medical University, Shanghai 200433, China.

**Keywords:** Gastric cancer, AQP5, gastric cancer stem cells, autophagy

## Abstract

Aquaporin 5 (AQP5) has been shown to have a pro-carcinogenic effect in numerous types of malignancies. This research intends to investigate the role and the molecular mechanism of AQP5 on enriched gastric cancer stem cells (GCSCs).

**Methods**: Immunohistochemistry, western blot (WB), and RT-qPCR techniques were employed to identify the presence of AQP5 in gastric cancer (GC) and the neighboring paracancerous tissues. Additionally, a statistical analysis was conducted to determine the correlation between AQP5 expression and the pathological and histological parameters. Furthermore, the study aimed to assess the predictive value of AQP5 expression in long-term survival after GC surgery. GCSCs were enriched using the serum-free culture method. The expression of AQP5 in enriched GCSCs was explored using RT-qPCR and WB. Plate cloning, transwell, WB, RT-qPCR, and the sphere-forming assay were utilized to monitor the proliferation, migration, and self-renewal capability of GCSCs after AQP5 knockdown. WB and Immunofluorescence for Detecting the Effect of AQP5 on Autophagy. WB, RT-qPCR, and other experiments were used for in-depth investigation of the potential molecular regulatory mechanism of AQP5 in GC.

**Results**: AQP5 was highly expressed in GC tissues and GC cells, and overexpression of AQP5 was associated with lymph node metastasis, increased tumor size, and low 5-year postoperative survival in GC patients; other studies have shown that the AQP5 was highly expressed in GCSCs. Knockdown of AQP5 suppressed tumorigenesis *in vivo* and inhibited the proliferative, migratory, and self-renewal capability of GCSCs. It was also found that AQP5 could activate the autophagy phenomenon of GCSCs, and mechanistically, we found that AQP5 could regulate TRPV4 to affect the self-renewal ability of GCSCs.

**Conclusion:** AQP5 can be further explored for GC therapy, as it has shown a significant impact on the self-renewal capability of GCSCs, which prevents GC progression.

## Introduction

Gastric cancer (GC) ranks as the fifth most prevalent malignant neoplasm on a global scale, with the third highest fatality rate 1. In China, GC is the third most common cancer type and has consequently risen to become the second primary cause of cancer-related fatality 2. Several reports have shown evidence of the presence of cancer stem cells (CSCs) within solid tumors, including GC 3. Comparable to normal stem cells, CSCs tend to self-renewal and have the potential to develop into a heterogeneous population of tumor cells, hence contributing to the accelerated proliferation of tumors 4. The CSCs are commonly referred to as "tumor-initiating cells", and it has been observed that a minimal number of CSCs have the ability to initiate tumorigenesis 5. The process known as epithelial-to-mesenchymal transition (EMT) leads to a reduction in the levels of adhesion molecules, including E-cadherin, in CSCs. This decrease in adhesion molecules facilitates the occurrence of tumor metastasis, making CSCs a significant factor in promoting metastatic spread 6. Additionally, CSCs are recognized as a potential cause of tumor recurrence and serve as "seed cells" for tumor recurrence 7. Moreover, the presence of CSCs is also a significant factor contributing to the development of resistance to radiation in tumors during radiotherapy 8. It is evident that CSCs play a pivotal role in the progression of tumors, and the strategic targeting of CSCs holds significant potential in the prevention and management of GC. Further exploration is required to investigate novel compounds that specifically target CSCs in GC, as well as their underlying regulatory processes.

In recent years, it has been found that the expression of water channel proteins is closely related to the migration and metastasis of a variety of malignant tumor cells. AQP5 is one of the most important members of the water channel protein family, and a significant upregulation of AQP5 has been observed in many malignant neoplasms, including gastric carcinoma 9. The literature reports that AQP5 has been identified as a potential target for inhibiting breast cancer growth 10. Additionally, the literature has proposed that AQP5 expression is elevated in intestinal-type gastric adenocarcinomas compared to normal tissues 11, and this upregulation contributes to the progression of GC. However, the precise functions of AQP5 in the development of gastric carcinoma, including the specific target cells and molecular mechanisms involved in its regulation, remain unexplored.

In summary, this work examined the regulatory effects of AQP5 on the activity of GCSCs, intending to offer new approaches and concepts for the diagnosis and treatment of GC.

## Materials and Methods

### Materials

The human GC cell lines (SGC-7901, MKN-45, BGC-803, AGS, and HGC-27) and gastric normal cells GES-1 were obtained from the cell bank of the Chinese Academy of Sciences in Shanghai, China. Wuhan Hanheng Biological Company constructed the AQP5 lentivirus. The antibodies used in this study included rabbit anti-AQP5 (Cell Signaling Technology, USA), rabbit anti-CD44, OCT4, CD133, β-actin, P62, and LC3 (proteintech, China), rabbit anti-TRPV4 (Affinity, China). The secondary antibody used was horseradish peroxidase-coupled goat-anti-rabbit (LiankeBio, China). The cell culture media used were RPMI-1640 medium, DMED/F12 medium, and B27 medium (Gibco, USA). The protein quantification kit used was the bicinchoninic acid (BCA) protein quantification kit (Beyotime Biotechnology, China). The glass dishes used were from Biosharp (China), and the transwell chambers used were from Corning (USA). The Epidermal Growth Factor (EGF) and Basic Fibroblast Growth Factor (bFGF) were acquired from PeproTech (USA). The serum was obtained from Eexell (Uruguay). The FastPure Cell/Tissue Total RNA Isolation Kit and ChamQ Universal SYBR qPCR Master Mix Kit were purchased from Vazyme (Nanjing, China).

### Samples

The clinicopathological data of the patients were obtained from the electronic case system and case management system of the First Affiliated Hospital of Bengbu Medical University. A total of 73 patients who had undergone radical surgery for GC in our hospital between January 2017 and December 2017 were included in the analysis. Among them, 55 were male and 18 were female. The age of the patients ranged from 34 to 79 years, with a median age of 63 years. The analysis primarily consisted of the patient's hospitalization number, pathology number, name, gender, age at the time of surgery, date of surgery, TNM stage, depth of tumor invasion, site of lesion, lymph node metastasis, distant metastasis, pathological staging, degree of tumor differentiation, tumor size, and report cards from relevant laboratory and imaging examinations. Telephone follow-up, as well as querying outpatient and inpatient system review records, were utilized to determine the patients' postoperative survival and whether or not tumor-related deaths happened 5 years after surgery. The subsequent period concluded in October 2023. Overall survival (OS) of patients was defined as the period from the date of surgery until the end of the follow-up period or the occurrence of death. The Ethics Committee of the First Affiliated Hospital of Bengbu Medical University examined and approved the study, and all subjects completed an informed consent form (Ethics Approval Number: 2023YJS284).

### Handling of samples

The tissue wax block specimen consists of GC radical surgery resection specimens that were fixed in formalin and sent to pathology. These specimens were then routinely taken for paraffin embedding and preserved at room temperature. The GC tissues and corresponding paracarcinoma tissue wax blocks were retrieved from the Department of Pathology based on the patient's pathology number. These wax blocks were used for immunohistochemical staining of AQP5 and CD133 in the GC surgical tissue. Tissue specimens from newly formed GC lesions and corresponding normal gastric mucosa (at least 5cm away from the tumor margin) were taken immediately after gastrectomy during GC surgery. Following collection, the specimens were washed with saline solution to eliminate any blood stains on the surface. They were then dissected using scissors and transferred into sterile EP tubes that were free of enzymes. The tubes were promptly frozen and stored in a refrigerator set at -80 °C for future analysis using immunoblotting WB and reverse transcription RT-qPCR to detect AQP5 in the tissues.

### Immunohistochemical staining

Pathological wax blocks were cut into 4 μm thin slices using a pathology sectioning machine, and the slices were placed in an oven at 65 °C for 1 h. Xylene was deparaffinized and then alcohol was sequentially hydrated, and citrate buffer was used to repair the antigen. 5% BSA or closed serum was added dropwise to the slices and incubated in a thermostat at 37 °C for 30 min. PBS was used in place of primary antibody for immunostaining blanks, non-immunized goat or rabbit serum was used for negative controls, and lung tissue was used for positive controls. These pretreated slides were incubated at 4 °C overnight with antibodies against AQP5 (1:100 dilution), CD133 (1:50 dilution), followed by incubation with the HRP-conjugated secondary antibody. DAB was used to develop the color, followed by restaining using hematoxylin.

### Evaluation of immunohistochemical staining

Cells with yellowish-brown staining granules were positive cells, and three observation areas were randomly selected for each pathology section under a 400× microscope, and it was ensured that the selected observation areas were appropriately chosen to avoid positive expression of hemorrhage, necrosis, and cells at the edge of the interface as much as possible (positive staining of such cells was mostly endogenous or artificially interfered). The results of section staining were scored by two pathologists on the percentage of positive cells and staining intensity, respectively, with a score ranging from 0-12, ≥ 9 being high expression and < 9 being low to moderate expression, and the product of the two scores was calculated as the score of the slice, and the scores obtained by the two pathologists were averaged.

### Cell culture

The RPMI-1640 media supplemented with 10% fetal bovine serum and 1% penicillin-streptomycin was used to culture the human GC cell lines AGS, BGC-803, MKN45, SGC-7901, HGC-27, and gastric normal cell GES-1. The cell cultures were maintained in a controlled environment in a cell culture incubator at a temperature of 37 °C and a CO_2_ concentration of 5%. GCSCs were cultured in ultra-low-absorption six-well plates. The experiment involved seeding 1 × 10^3^ cells per well in six-well plates. The cells were cultured in a serum-free suspension medium consisting of DMEM/F12 basal medium supplemented with B27 (10 μL/mL), bFGF (10 ng/mL), and EGF (20 ng/mL). Each well contained 2 mL of the culture system. For 2-3 days, 1 mL of serum-free medium was replenished periodically. After one week of culture, the cells were either passed on or utilized for experimental purposes. After 7 days, the dimensions and quantity of spheroplasts were recorded.

### Extraction of total RNA and RT-qPCR

The FastPure Cell/Tissue Total RNA Isolation Kit was utilized to extract total RNA, which was subsequently reverse-transcribed into complementary DNA (cDNA). About 1 µg of total RNA was subjected to RT-qPCR using ChamQ Universal SYBR qPCR Master Mix Kit. Primer upstream sequence of AQP5: GCCACCTTGTCGGAATCTACT, primer downstream sequence: CCTTTGATGATGGCCACACG. Primer upstream sequence of CD44: CCTCTTGGCCTTGGCTTTG, primer downstream sequence: CTCCATTGCCACTGTTGATCAC. Primer upstream sequence of OCT4: GGACCCAGGGAGAGACGTAA, primer downstream sequence: CAUGGAUUUUUUGGAGCAGG. Primer upstream sequence of TRPV4: TCAATGAACTCTGCTGGGGGACAAG, primer downstream sequence: TGGTAGTAGGCGGTGAGAGTGAAG. Primer upstream sequence of CD133: GCTTTGCAATCTCCCTGTTG, primer downstream sequence: GCTTTGCAATCTCCCTGTTG. Primer upstream sequence of β-actin: GACCTGTACGCCAACACAGT, primer downstream sequence: CTCAGGAGGAGCAATGATCT. Amplification was performed in a 20 µL reaction at 95 °C for 5 min, followed by 40 cycles of 95 °C (10 s), 60 °C (30 s), 95 °C (15 s), 60 °C (60 s), and 95 °C (15 s) on a LightCycler 96 real-time PCR System (Roche, Basel, Switzerland). The findings were determined via the 2^-△△CT^ approach.

### Western blot

The cells from each group were collected and subjected to lysis using a lysis solution (RIPA and PMSF at a ratio of 100:1). The lysis process involved shaking on ice, with shaking repeated every 10 min, for a total of 3 times. Following lysis, the protein content was quantified using the BCA method. Afterward, the proteins were separated using sodium dodecyl sulfate-polyacrylamide gel electrophoresis (SDS-PAGE) and then transferred onto a polyvinylidene fluoride (PVDF) membrane. The PVDF membrane was incubated with milk at room temperature for a duration of 1-2 h. Following that, primary antibodies (AQP5, OCT4, CD44, P62, LC3, TRPV4, and CD133) were added in a ratio of 1:1000. The primary antibody was then incubated overnight at 4 °C using a shaker. The next day, the membrane was incubated with a secondary antibody (horseradish peroxidase coupled to goat anti-rabbit) at a ratio of 1:5000 for a period of 2 h at room temperature. The membrane was then washed three times with tris-buffered saline with 0.1% Tween® 20 detergent (TBST) solution. Finally, the protein bands were developed using enhanced chemiluminescence (ECL) and eventually detected. The presence of protein bands was observed.

### Gene Expression Profiling Interactive Analysis

The GEPIA database, accessible at https://gepia.cancer-pku.cn, is an open-access platform that offers comprehensive RNA sequencing data for several types of cancer. The utilization of GEPIA assessed the relationship between AQP5 and stemness parameters in stomach adenocarcinoma (STAD). Genes that interact with AQP5 were analyzed using the STRING (http://string-db.org) database.

### Plate cloning experiment

The cells were digested with trypsin, counted (1 × 10^3^ cells/well), and added to a six-well plate. The state of cells was consistently monitored, nutrients were timely replenished, and the culture medium was changed as needed. Following a period of two weeks, the fixation was performed using a 4% paraformaldehyde solution (15 min). Next, the cells were subjected to staining with crystalline violet for an additional 15 min. Following the staining process, the cells were rinsed with phosphate-buffered saline (PBS) and counted.

### Transwell experiment

Trypsin was used to detach cells and obtain a single-cell suspension. A volume of 200 μL of basal medium, containing 2 × 10^4^ cells, was introduced into the upper chamber of a transwell chamber. In the lower chamber, 750 μL of serum-containing medium with a serum concentration > 15% was added. After 24 hours, the upper chamber was rinsed with PBS, and the cells within the top chamber were immobilized using a 4% paraformaldehyde solution and stained with crystal violet for 15 min. After the chambers were dried, the cells were enumerated using an inverted microscope.

### Sphere formation assay

The cells were trypsinized, enumerated, and subsequently seeded into ultra-low adsorption six-well plates. The plates were filled with serum-free suspension culture medium, and cells at a concentration of 200 cells/well were added. The serum-free suspension medium used in this experiment was composed of DMEM/F12 basal medium supplemented with B27 (10 μL/mL), bFGF (10 ng/mL), and EGF (20 ng/mL). The serum-free media (1 mL) was replenished every 2-3 days in each 2 mL culture well. The cells were cultured for one week, either to allow for cell passage or utilization in experimental procedures. After 7 days, the size and quantity of spheres were examined and quantified.

### Lentiviral transfection and siRNA transfection

Wuhan Hanheng Biological Co. Ltd. manufactured recombinant lentiviral vectors that carried human AQP5 knockdown or overexpression gene. These vectors were transfected, and the stably transfected strains were obtained by puromycin screening. The interference sequences were:

sh-NC-F:5'-GATCCGTTCTCCGAACGTGTCACGTAATTCAAGAGATTACGTGACACGTTCGGAGAATTTTTTC-3', sh-NC-R:5'-AATTGAAAAAATTCTCCGAACGTGTCACGTAATCTCTTGAATTACGTGACACGTTCGGAGAACG-3'. sh-AQP5-F:5'-GATCCGCCATCCTTTACTTCTACCTGCTCTTTCAAGAGAAGAGCAGGTAGAAGTAAAGGATGGCTTTTTTG-3', sh-AQP5-R:5'-AATTCAAAAAAGCCATCCTTTACTTCTACCTGCTCTTCTCTTGAAAGAGCAGGTAGAAGTAAAGGATGGCG-3'.

The si-TRPV4 interfering fragment was synthesized by Gemma Shanghai, and the interfering sequence was as follows:

si-NC-F:5'-UUCUCCGAACGUGUCACGUTT-3', si-NC-R:5'-ACGUGACACGUUCGGAGAATT-3'. si-TRPV4-F:5'-GCUGGAUGAAUGCCCUUUATT-3', si-TRPV4-R:5'-UAAAGGGCAUUCAUCCAGCTT-3'.

### Immunofluorescence

The single-cell suspension was planted into confocal petri dishes. Following the cells' attachment to the wall, they were fixed for 15 min with pre-cooled methanol, after which 0.3% Triton X-100 was used to permeate the membrane for 20 min. Followed by three times rinsing with PBS. After an hour of incubation with 1% bovine serum albumin (BSA), the cells were subjected to thrice PBS washes and an overnight incubation at 4 °C with the primary antibody, followed by incubation with the secondary antibody. After washing, as previously mentioned, DAPI staining was performed for 2 min.

### *In vivo* animal xenograft experiments

A group of female nude mice, aged 4 weeks, was subjected to random allocation into two groups, each consisting of 5 animals. Subsequently, both groups were administered a subcutaneous injection containing 2 × 10^6^ GCSCs. The research study received ethical approval from the Ethics Committee of Bengbu Medical university with reference number 2023.547.

### Statistical analysis

Image J, Excel, SPSS27, and GraphPad Prism 8 software were used to analyze the data statistically, and the experimental data were repeated more than 3 times. An independent samples t-test was conducted to compare the differences between the two groups. One-way analysis of variance (ANOVA) was utilized to compare different groups. A significance level of P < 0.05 was used to determine whether there was a statistically significant difference in the results across the groups.

## Results

### Expression of AQP5 in GC tissues and paracancer tissues

**Figure 1:**
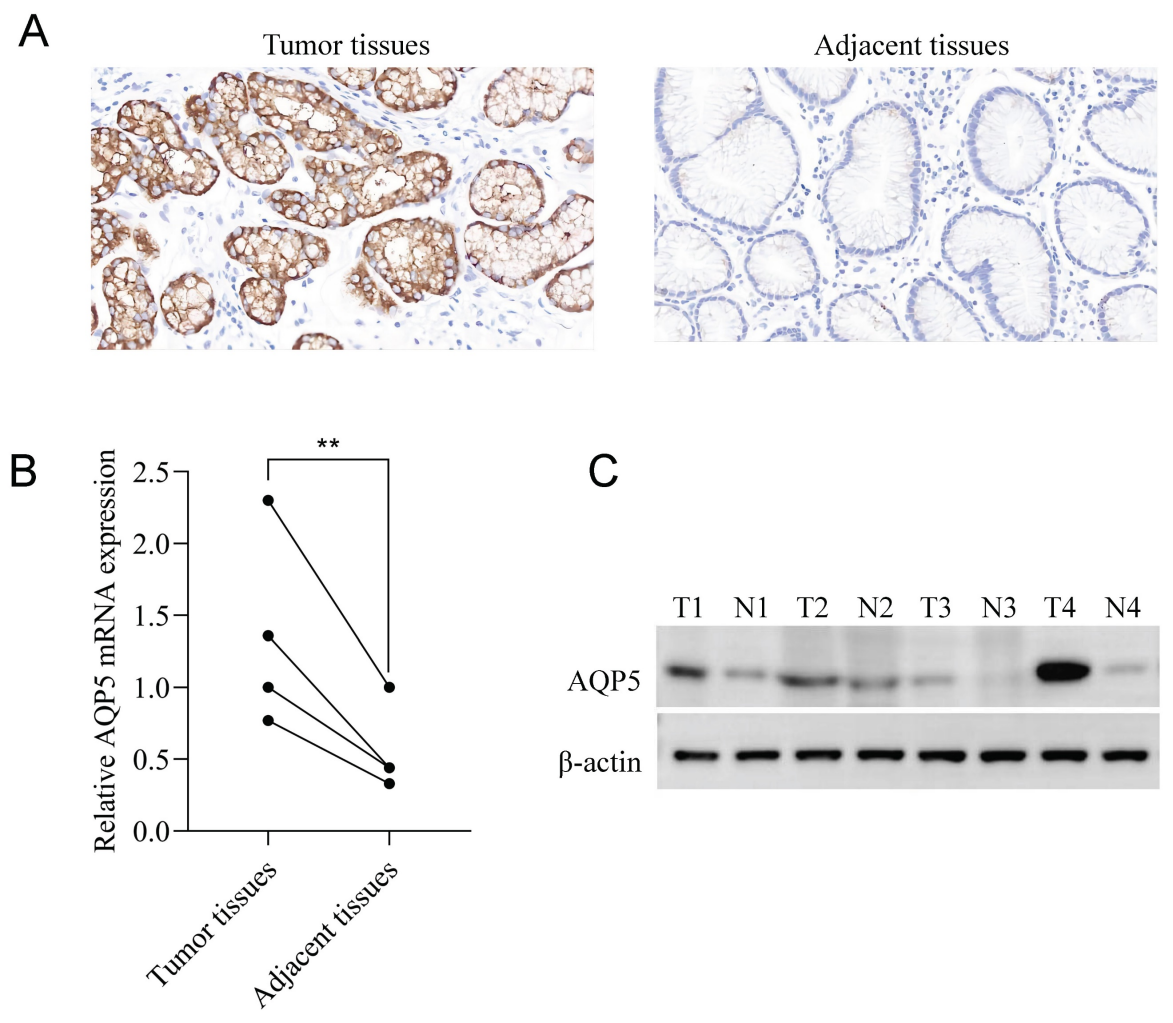
** Expression of AQP5 in GC tissues and paracancer tissues. (A)** Immunohistochemical staining showing the expression of AQP5 in GC tissues and paracancerous tissues. Magnification, ×400. **(B, C)** Expression of AQP5 in GC tissues and paracancerous tissues by WB, and RT-qPCR. **P < 0.01.

Immunohistochemical staining results showed that AQP5 was highly expressed in 46/73 (63%) and low expressed in 27/73 (37%) GC patients, while it was not expressed or weakly expressed in the corresponding paracancerous tissues, and it was mainly expressed in the cytomembrane and cytoplasm of the GC cells (Fig. 1A). Fluorescence quantitative RT-qPCR and WB results showed that the expression of AQP5 in GC tissues was higher than that in paracancerous tissues (P < 0.01) (Fig. 1B-C).

### Relationship between AQP5 expression and clinicopathologic parameters

The patients were categorized into two groups based on the expression of tissue AQP5: the AQP5 low expression group (n=27, 37.0%) and the high expression group (n=46, 63.0%). Table 1 demonstrates a notable disparity in the expression of AQP5 between GC tissues and lymph node metastasis (P < 0.01), as well as tumor size (P < 0.05). However, the expression of AQP5 does not correlate with various pathological parameters in GC patients, including age, gender, degree of tumor cell differentiation, TNM staging, depth of infiltration, lesion site, and neuropil invasion (Table 1).

### High expression of AQP5 in GC tissues is associated with poor prognosis

**Figure 2:**
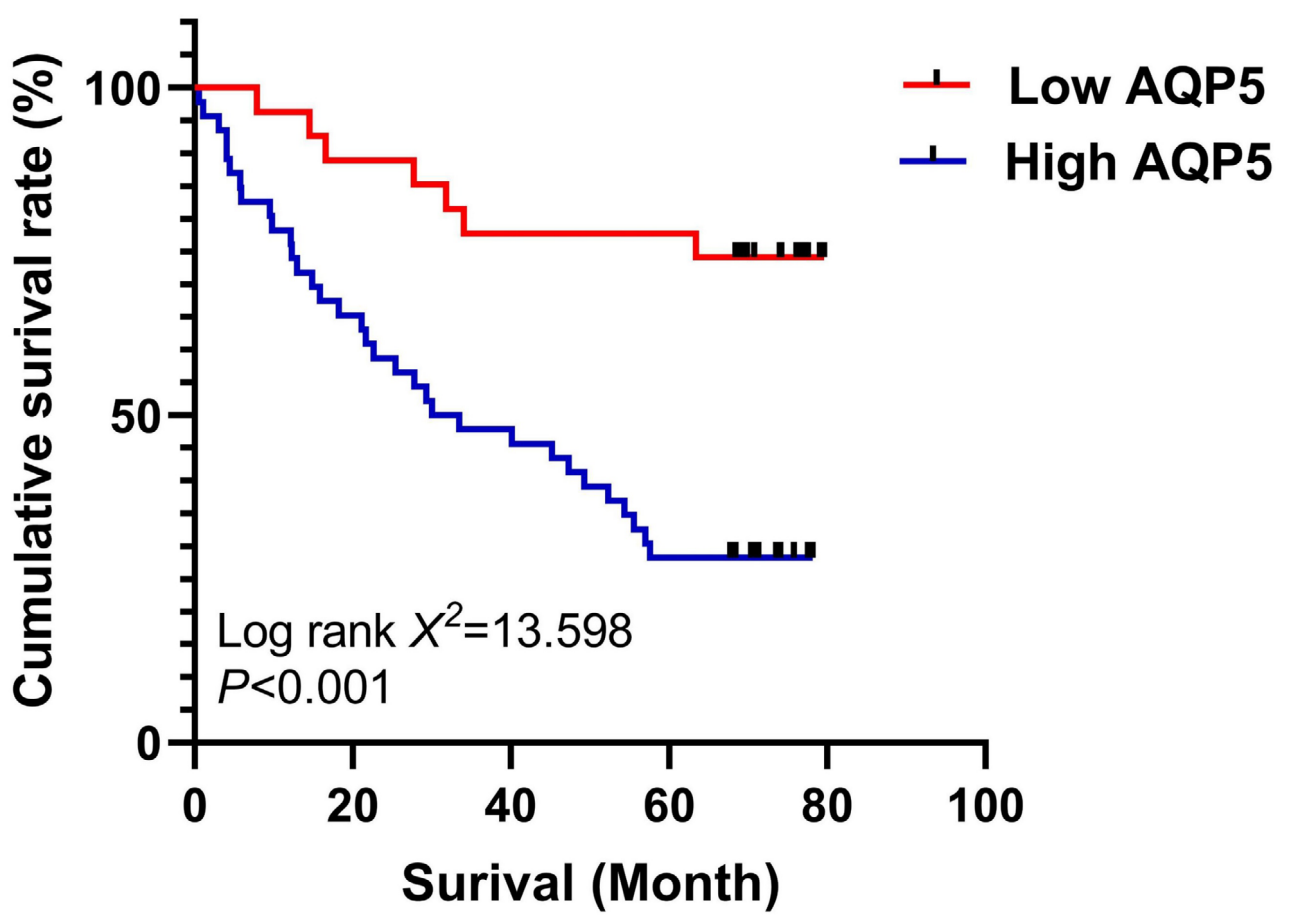
** Survival curves using the Kaplan-Meier method by Log-rank test**: The patients with high AQP5 expression had shorter overall survival than those with low AQP5 expression. ***p < 0.001.

The relationship between AQP5 expression in GC tissues and 5-year survival after radical GC surgery was analyzed using the K-M survival curve, Log-Rank analysis showed that high expression of AQP5 (Fig. 2) in GC tissues of GC patients' tissues was associated with low postoperative survival of GC patients, (χ^2^=13.598, P < 0.001). Univariate analysis showed that high AQP5 expression, TNM stage, lymph node metastasis, and neurovascular invasion were risk factors affecting 5-year survival after radical GC surgery, and multifactorial Cox regression modeling analysis showed that high AQP5 expression, as well as neurovascular invasion, could be used as an independent risk factor affecting 5-year survival after radical GC surgery as shown in Table 2.

### Correlation between AQP5 and CD133 expression in GC tissues

**Figure 3:**
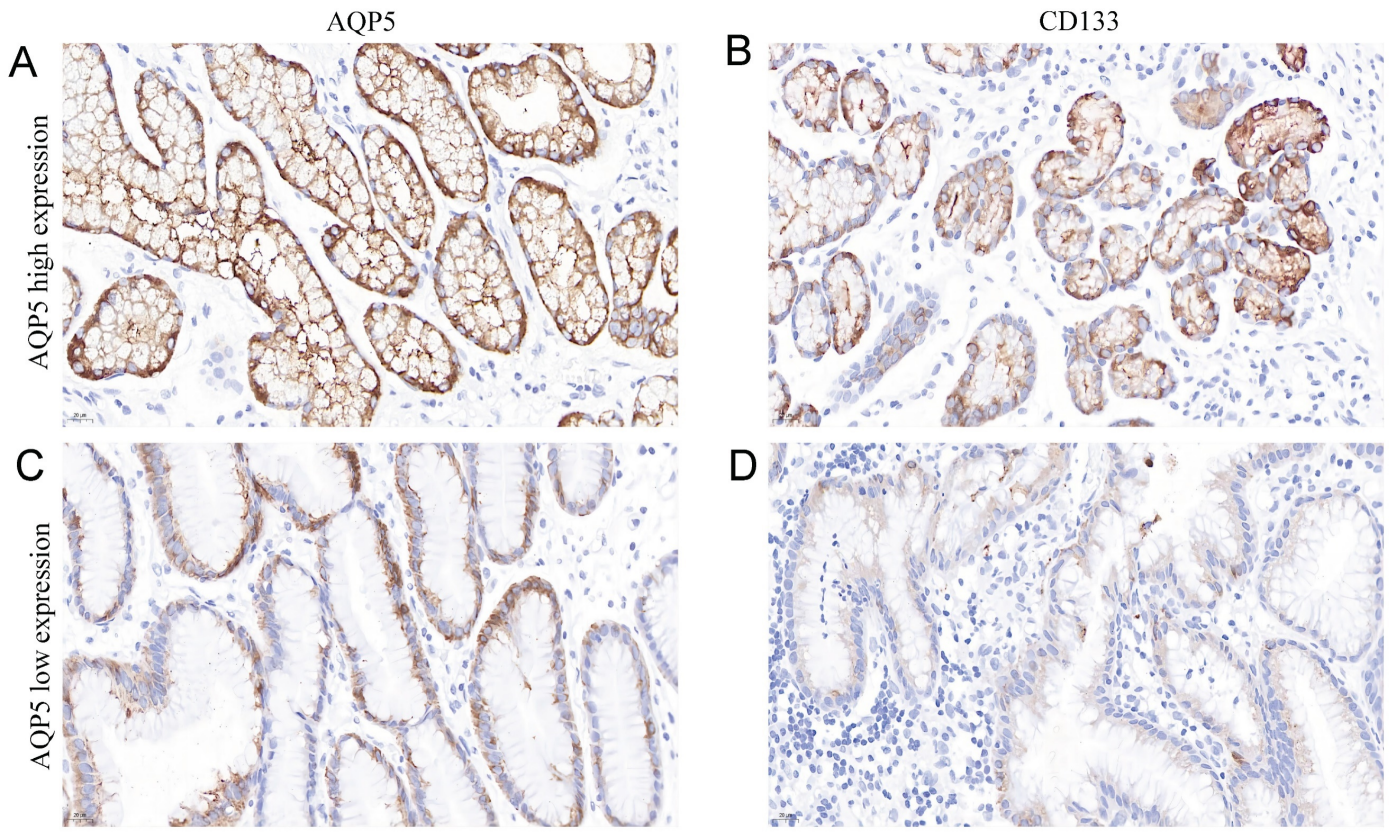
** Representation images of AQP5 and CD133 expression in GC tissues by immunohistochemical staining. Magnification, ×400**. (**A-B**) High AQP5 expression in gastric adenocarcinoma with positive CD133 expression. (**C-D**) low AQP5 expression in gastric adenocarcinoma with negative CD133 expression.

It is well known that CD133 is a classical GC stem cell marker, and we investigated the correlation between the expression of AQP5 and GC stem cell marker CD133 in GC tissues by immunohistochemical analysis, as shown in Table 3 and Figure 3, the expression level of AQP5 was positively correlated with the expression level of CD133 (r = 0.311, P < 0.01).

### Isolation and characterization of GCSCs

**Figure 4:**
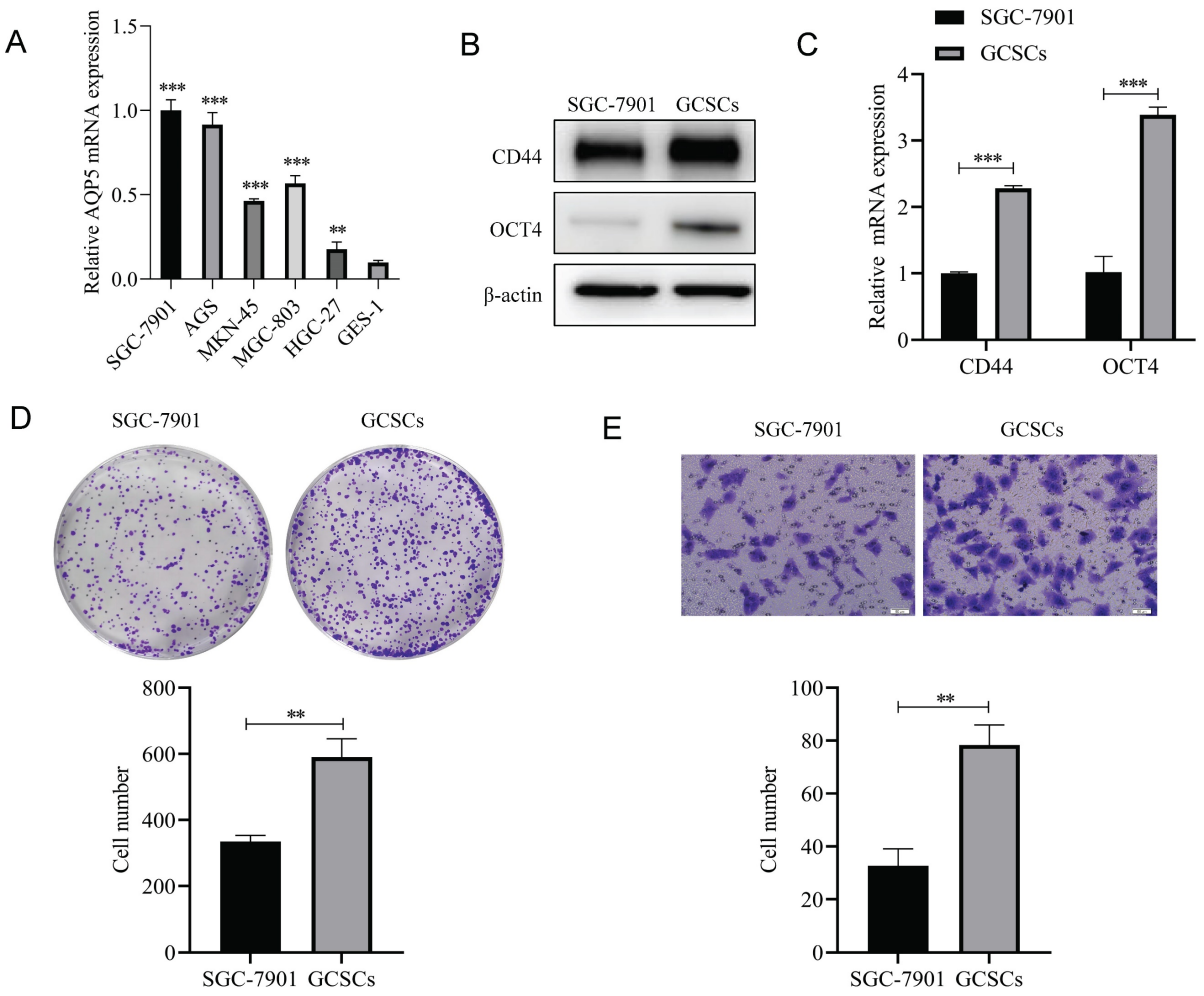
** Isolation and characterization of GCSCs. (A)** mRNA expression levels of AQP5 in five GC cell lines SGC-7901, AGS, MKN45, BGC-803, and HGC-27 and gastric normal cell GES-1 by RT-qPCR. **(B, C)** WB and RT-qPCR to detect the mRNA and protein expression levels of CD44 and OCT4 in SGC-7901 cells and the serum-free enriched GCSCs.** (D)** Plate cloning assay to detect the proliferation ability of SGC-7901 cells and their enriched GCSCs. **(E)** Transwell assay to detect the migration ability of SGC-7901 and the enriched GCSCs. **p < 0.01, ***p < 0.001.

Firstly RT-qPCR results showed that AQP5 was highly expressed in five GC cell lines (SGC-7901, AGS, MKN45, BGC-803, and HGC-27) compared to gastric normal cells GES-1 cells (Fig. 4A), and we selected SGC-7901, which had a relatively high expression of AQP5, as a follow-up experiment. GC cell (SGC-7901) were enriched with GCSCs by serum-free culture. The expression of GC stemness markers (CD44, OCT4) was detected by WB and RT-qPCR. The outcomes obtained from WB and RT-qPCR analyses demonstrated that the GCSCs enriched by serum-free culture exhibited a notable upregulation in the expression of CD44 and OCT4 as compared to SGC-7901 cells (Fig. 4B-C). Some studies have reported that GCSCs cultured in serum-free suspension have stronger proliferation and migration ability 12, which was also demonstrated in the present experiment. The findings obtained from plate cloning and transwell experiments demonstrated that the serum-free culture enrichment of GCSCs led to enhanced proliferation capabilities (Fig. 4D) and migration ability (Fig. 4E) compared with adherent cells. All of the above experimental results indicated that the enrichment of GCSCs was successful and could be used for subsequent experiments.

### High expression of AQP5 in GCSCs

**Figure 5:**
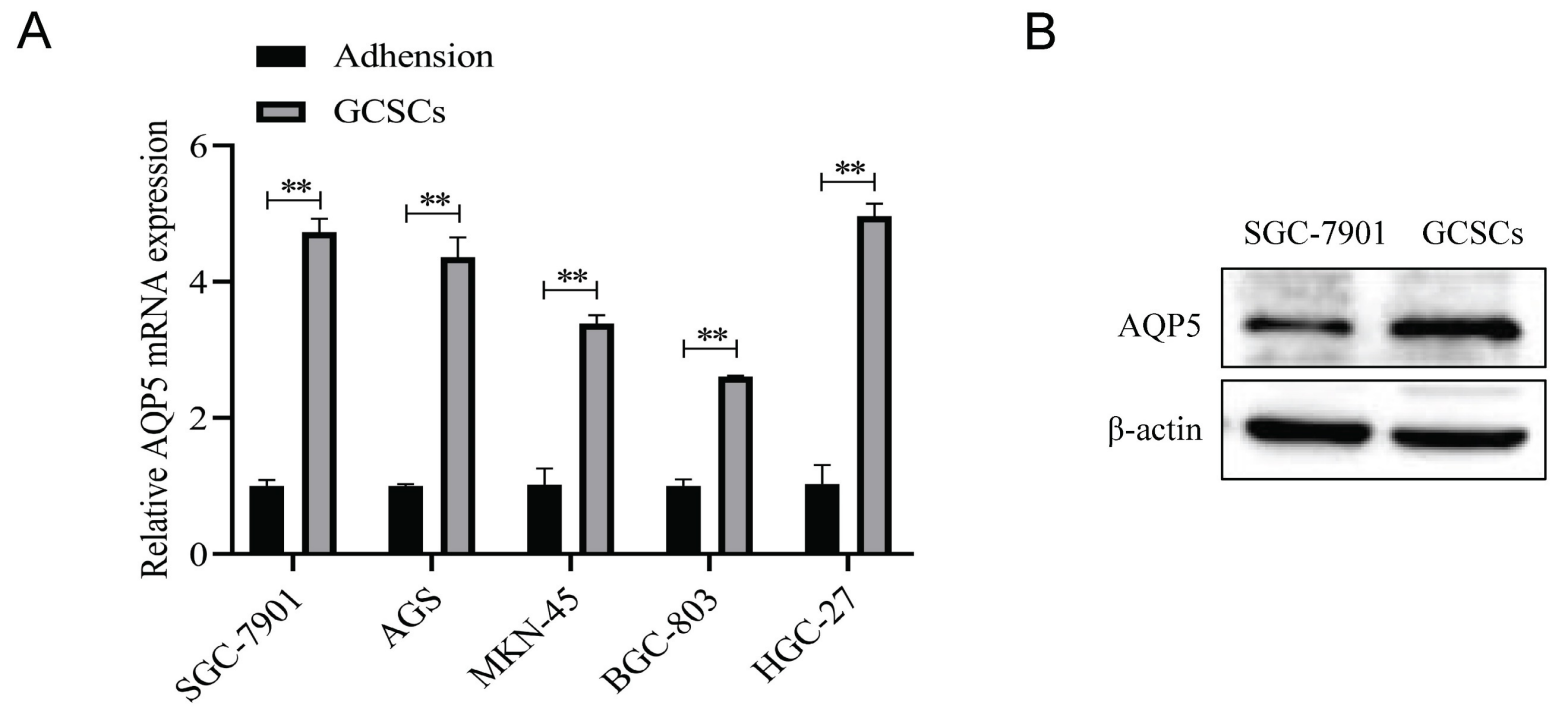
** Expression of AQP5 in GCSCs**. **(A)** AQP5 mRNA expression level was determined using RT-qPCR in five GC cell lines and GCSCs. **(B)** WB analysis revealed the protein expression level of AQP5 in SGC-7901 as well as GCSCs. **p < 0.01.

The mRNA expression levels of AQP5 in five GC cell lines, namely SGC-7901, AGS, MKN45, BGC-803, and HGC-27, together with the corresponding suspension-cultured GCSCs, were analyzed using RT-qPCR. The findings demonstrated that the expression of AQP5 in GCSCs was much higher compared to adherent cells cultivated under normal conditions (Fig. 5A). This was supported by WB experiments, which showed high expression of AQP5 in GCSCs compared to SGC-7901 cells (Fig. 5B).

### Knockdown of AQP5 inhibits the effects of proliferation, migration, and self-renewal capacity of GCSCs

**Figure 6:**
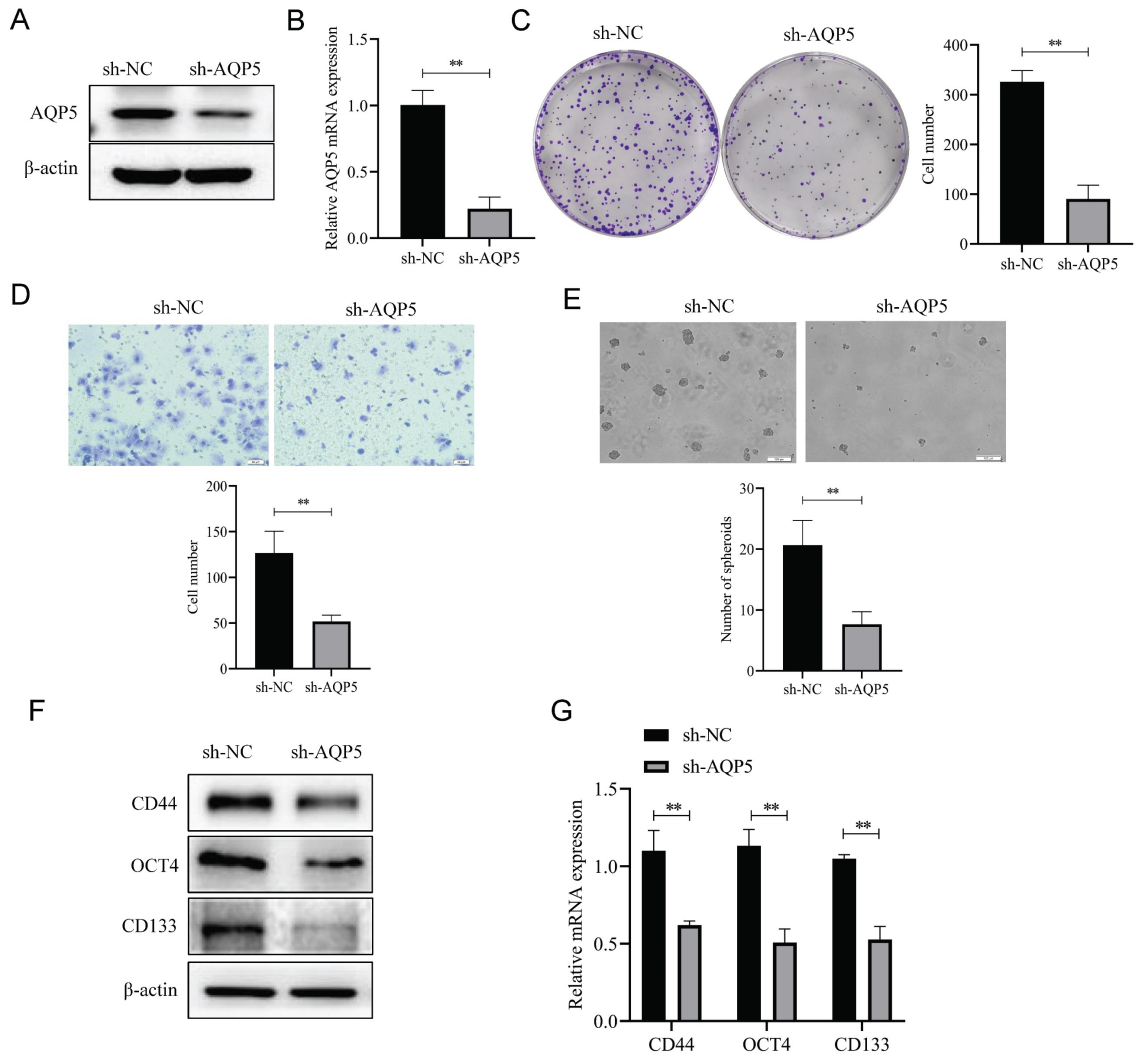
** Inhibition of AQP5 downregulation impeded GCSCs proliferation, migration, and self-renewal**. **(A, B)** Construction of knockdown AQP5 stabilized transplants in SGC-7901 cells. **(C)** Plate cloning assay analysis showed that the knockdown of AQP5 inhibited the proliferative ability of GCSCs enriched in SGC-7901 cells. **(D)** Transwell analysis revealed that AQP5 knockdown inhibited the migratory capability of GCSCs enriched with SGC-7901. **(E)** Using a ballooning assay, the capacity for self-renewal in AQP5-knockdown GCSCs enriched with SGC-7901 cells was determined. **(F, G)** WB and RT-qPCR analysis of stemness-associated proteins and mRNA levels in SGC-7901 cell-enriched GCSCs knocked down by AQP5. **p < 0.01.

To ascertain the regulatory function of AQP5 in GCSCs, stable transient cell lines were generated by knocking down AQP5 in SGC-7901 cells using lentiviral technology (Fig. 6A-B). Subsequently, the impact of this knockdown on the functionality of GCSCs was monitored. The findings of this work demonstrate that the inhibition of AQP5 expression has a suppressive effect on the proliferative capacity of GCSCs, as evidenced by the plate cloning results (Fig. 6C). From the transwell results, it was found that cell migration in GCSCs was inhibited after interfering with AQP5 (Fig. 6D). The sphere-forming assay revealed a reduction in the self-renewal capacity of GCSCs following interference with AQP5 (Fig. 6E). Additionally, the WB and RT-qPCR analysis demonstrated a downregulation in the expression of stemness markers CD44, CD133, and OCT4 in GC cells upon interference with AQP5 (Fig. 6F-G). Collectively, the findings suggest that the suppression of AQP5 expression hampers the proliferation, migration, and capacity for self-renewal in GCSCs.

### Overexpression of AQP5 increases GCSCs self-renewal ability and stemness marker expression

**Figure 7:**
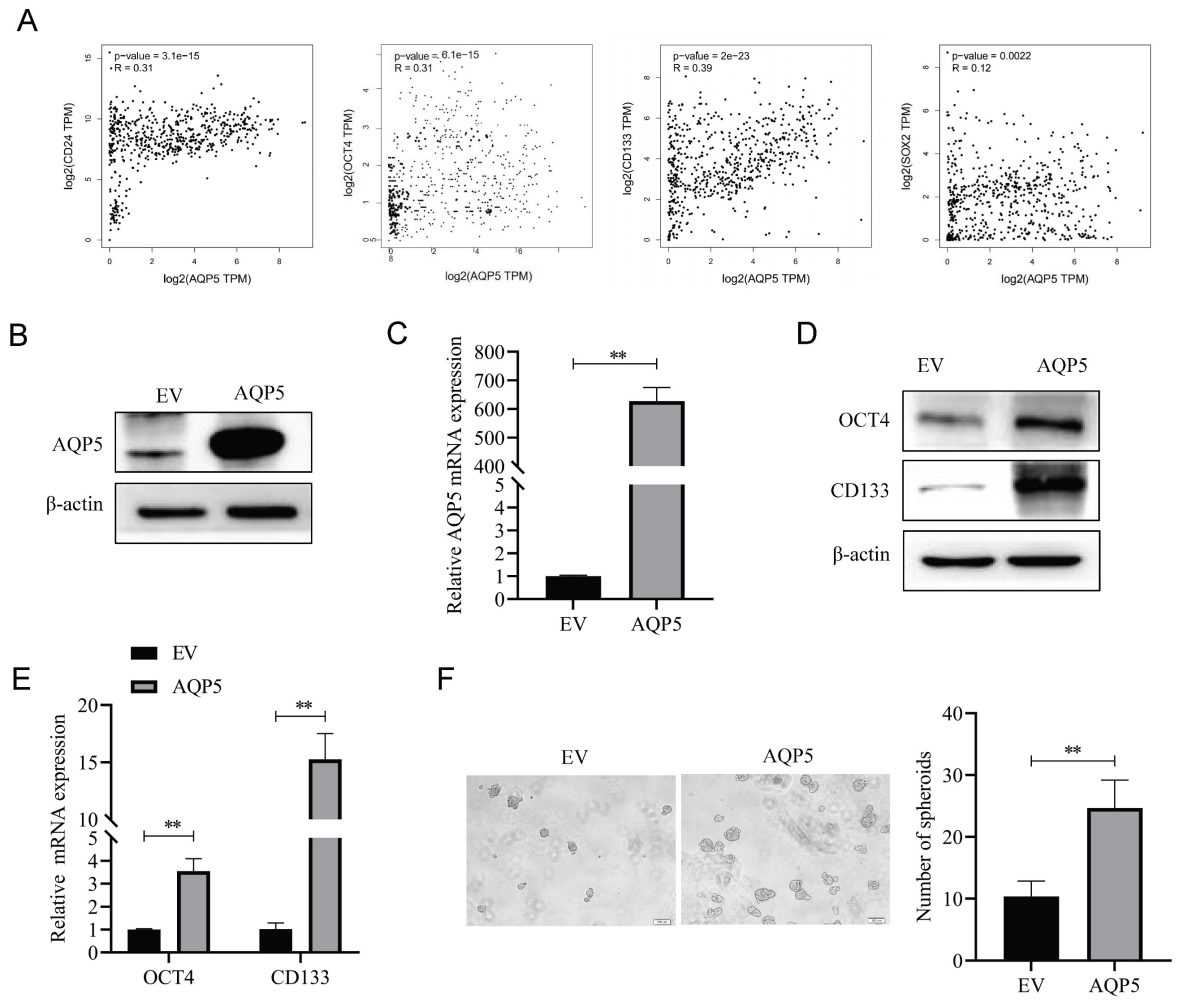
**Overexpression of AQP5 increased the self-renewal capacity of GCSCs**. **(A)** Correlation analysis of AQP5 with stemness-related markers in GC in the GEPIA database. **(B, C)** Results of WB and RT-qPCR showed successful construction of stable transplants with AQP5 overexpression. **(D, E)** Expression levels of stemness-associated markers in GCSCs enriched by AQP5 overexpression were analyzed by WB and RT-qPCR after the enrichment of stemness-associated markers in GCSCs by SGC-7901 cells. **(F)** The ballooning assay showed that AQP5 overexpression enhanced the ballooning capability of GCSCs. **p < 0.01.

To better understand the effect of AQP5 on the stemness characteristics of GCSCs, the relationship between AQP5 and stemness-associated factors was analyzed using the data of GC in the public database GEPIA (GEPIA-STAD). The findings demonstrate a substantial positive correlation between the expression levels of GC stemness markers CD133, OCT4, SOX2, and CD24 and the expression levels of AQP5 (Fig. 7A). To verify the result, stable transplants of AQP5 overexpression were constructed (Fig. 7B-C), and the expression of stemness-associated factors OCT4 and CD133 were detected using RT-qPCR and protein immunoblotting, respectively. The results revealed that overexpression of AQP5 significantly increased the mRNA and protein expression levels of stemness markers OCT4 and CD133 (Fig. 7D-E). The stem cell spheroidogenic experiment demonstrated that the overexpression of AQP5 enhanced the spheroidogenic capacity of GCSCs (Fig. 7F).

### AQP5 regulates GCSCs self-renewal through activation of autophagy

**Figure 8:**
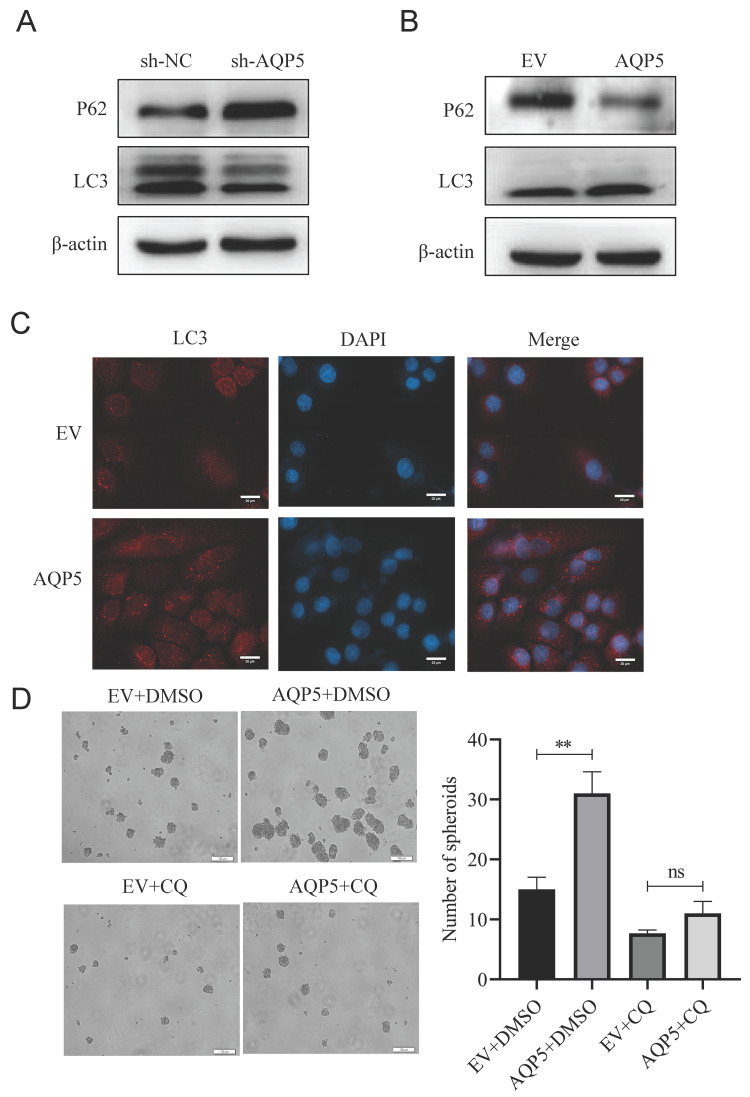
** AQP5 regulates the self-renewal capacity of GCSCs by activating autophagy**. **(A)** WB analysis of the effect of AQP5 suppression in SGC-7901 on autophagy-related protein expression in GCSCs. **(B)** The effect of AQP5 overexpression in SGC-7901 on the expression of autophagy-related proteins in GCSCs was determined via WB.** (C)** Confocal microscopy fluorescence images showed that overexpression of AQP5 in SGC-7901 induced the formation of autophagic vesicles.** (D)** Sphere-forming assay showed that chloroquine (CQ) reversed the sphere-forming ability of GCSCs overexpressing AQP5. **p < 0.01, ns: not significant.

According to reports, there exists a close association between AQP5 and the expression of the autophagy-related protein LC3 13. Based on this information, AQP5 may have a role in regulating the biological activities of GCSCs through the activation of autophagy. The experimental findings indicated that downregulating the expression of AQP5 in SGC-7901 cells resulted in a decrease in the expression of the autophagy-related protein LC3II and an increase in the expression of P62 (Fig. 8A). This suggests that the inhibition of AQP5 leads to a suppression of autophagy. Conversely, the overexpression of AQP5 in SGC-7901 cells reversed this effect (Fig. 8B). Additionally, the immunofluorescence results demonstrated that AQP5 induced an augmentation in autophagic vesicles (Fig. 8C). To further investigate this, the autophagy inhibitor chloroquine (CQ, 10 μmol/mL) was employed, which was observed to counteract the self-renewal ability of GCSCs induced by AQP5 (Fig. 8D). Based on the aforementioned findings, it may be inferred that the activation of autophagy by AQP5 has an impact on the self-renewal capacity of GCSCs.

### AQP5 activates autophagy in GCSCs by regulating TRPV4

**Figure 9:**
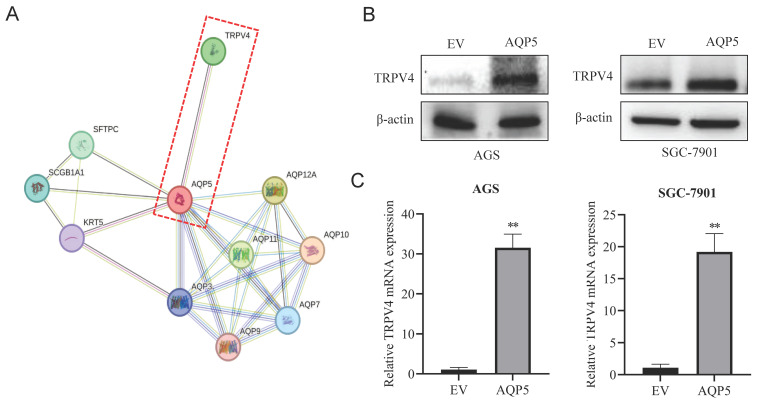

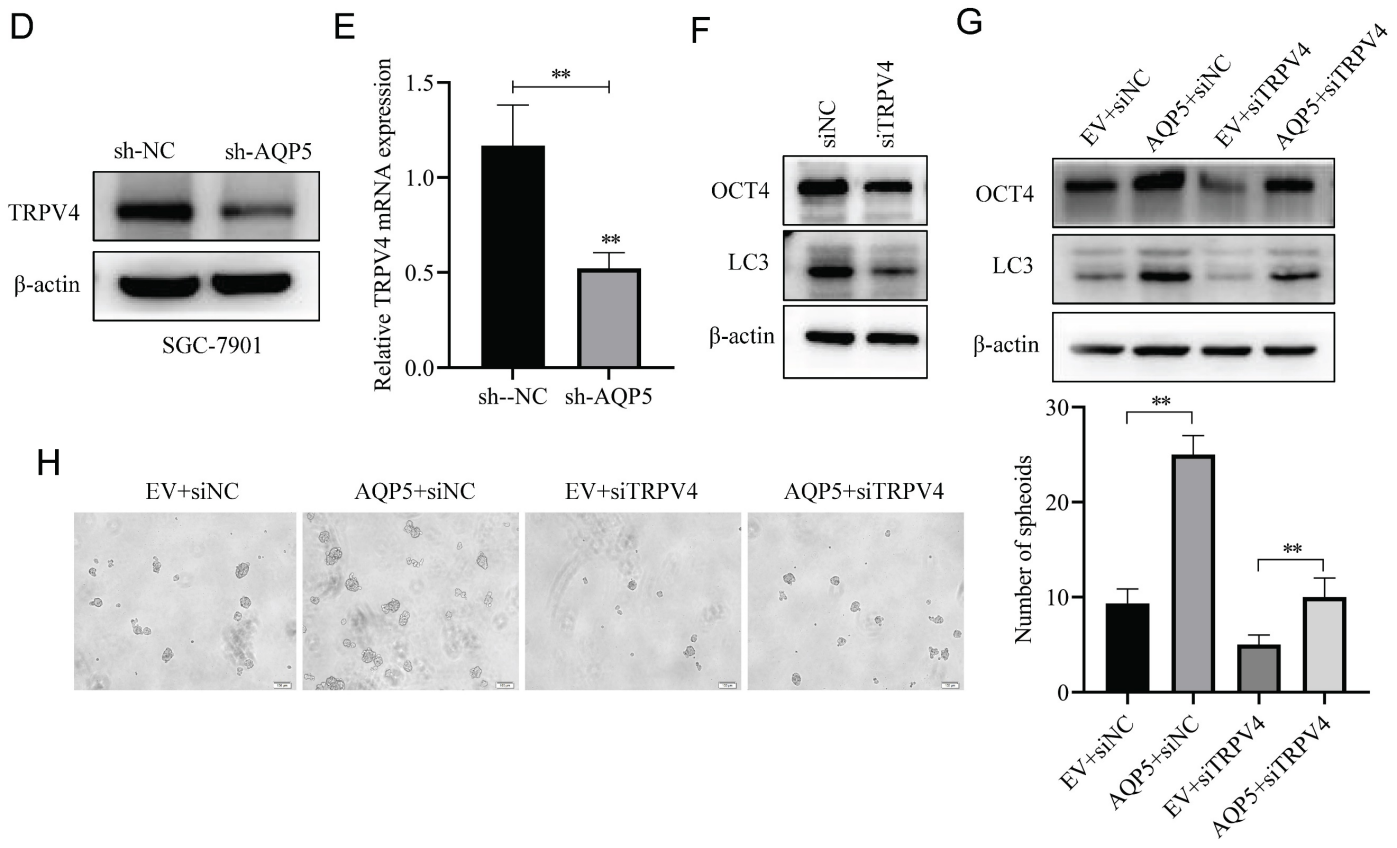
** Effect of AQP5-regulated TRPV4 on autophagy and self-renewal capacity of GCSCs**. **(A)** STRING protein database analysis of AQP5-related genes. **(B, C)** WB and RT-qPCR to detect mRNA and protein expression of TRPV4 after overexpression of AQP5 in SGC-7901 and AGS. **(D, E)** WB and RT-qPCR for mRNA and protein expression of TRPV4 after knockdown of AQP5 in SGC-7901. **(F)** Knockdown of TRPV4 in SGC-7901 significantly inhibited AQP5-induced protein expression of the stemness marker of GCSCs, OCT4, and autophagy protein LC3. **(G)** Knockdown of TRPV4 in SGC-7901 significantly inhibited AQP5-induced LC3 and OCT4 expression. **(H)** Knockdown of TRPV4 knockdown in SGC-7901 significantly inhibited AQP5-induced self-renewal. **P < 0.01.

This research discovered a direct protein interaction between AQP5 and TRPV4 on the STRING protein interactions website (Fig. 9A). To understand this relationship, we increased the expression of AQP5 in SGC-7901 and AGS cells. The results of WB and RT-qPCR experiments showed that overexpressing AQP5 led to an increase in TRPV4 mRNA and protein expression (Fig. 9B-C). However, when we reduced the expression of AQP5 in SGC-7901, it suppressed TRPV4 mRNA and protein expression (Fig. 9D-E). This suggests that AQP5 can regulate TRPV4 expression in GC. Subsequently, we examined the potential impact of AQP5-regulated TRPV4 on the stemness and self-renewal ability of GC cells. We discovered that TRPV4 expression knockdown in GCSCs enriched with SGC-7901 significantly suppressed the expression of the autophagy protein LC3 and the GC stemness marker OCT4 (Fig. 9F). Additionally, we observed that TRPV4 knockdown significantly suppressed the expression of autophagy protein LC3 and the GC stemness marker OCT4 in GCSCs induced by AQP5 (Fig. 9G), and the ball-forming capacity of AQP5-induced GCSCs was significantly suppressed (Fig. 9H).

### *In vivo* analysis of the regulation of GCSCs function by AQP5

**Figure 10:**
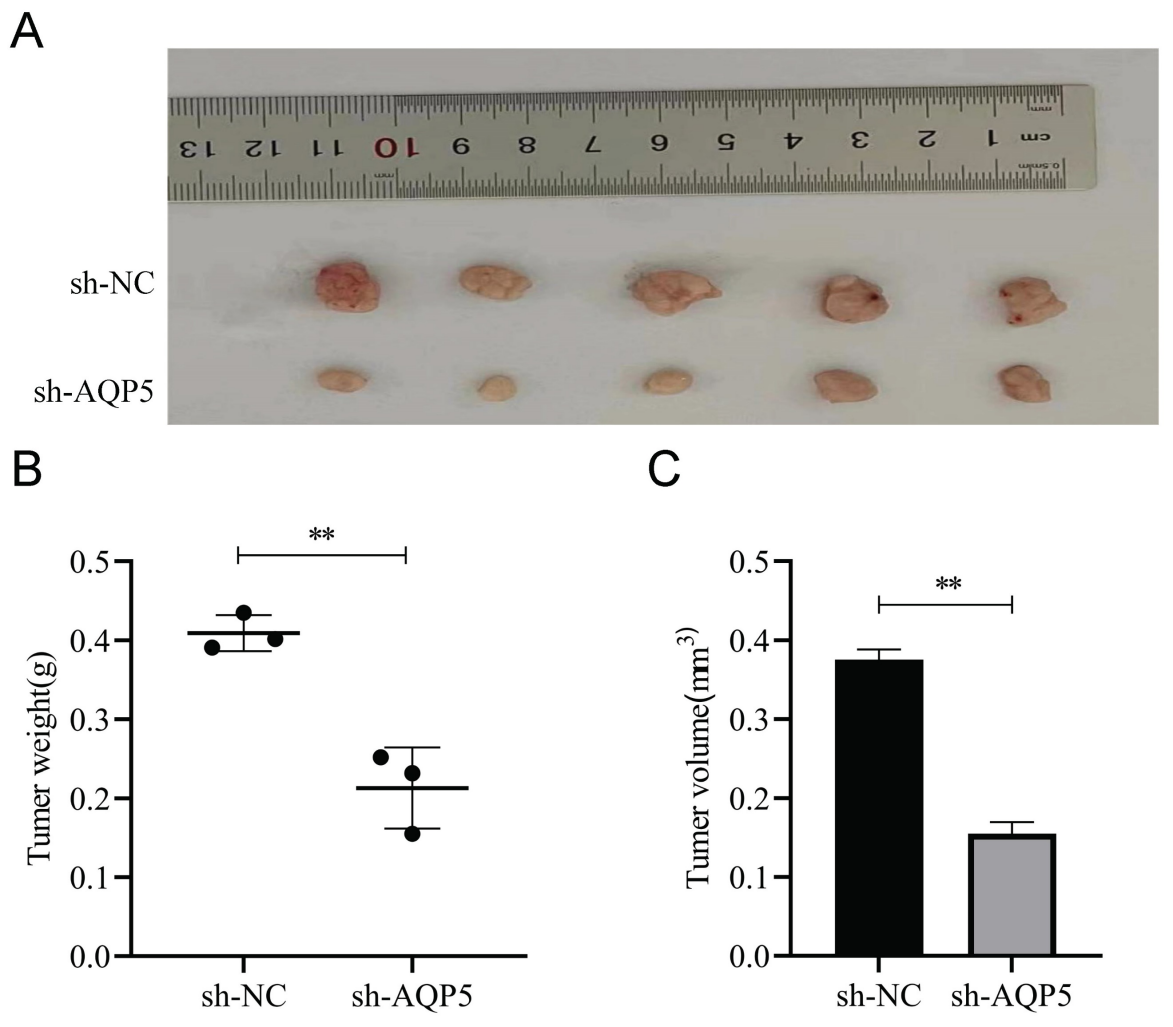
AQP5 inhibits tumor growth of GC *in vivo*. **(A)** Images depicting subcutaneous tumors extracted from nude mice. **(B, C)** Weight and volume of subcutaneous tumors excised from nude mice. **p < 0.01.

This work aimed to conduct a more comprehensive investigation of the impact of AQP5 on the formation of GC tumors in nude mice. GCSCs enriched by SGC-7901 cells with low expression of AQP5 were stabilized by subcutaneous injection of AQP5 in nude mice to establish xenogastric transplantation (Fig. 10A). The sh-AQP5 group exhibited reduced tumor weight and tumor volume in comparison to the NC group (Fig. 10B-C). The findings from *in vivo* investigations demonstrated that the inhibition of AQP5 expression resulted in a significant reduction in subcutaneous tumor development.

## Discussion

Multiple studies have demonstrated in recent years that GCSCs are the fundamental factor contributing to drug resistance, recurrence, and metastasis in GC 14, 15. The investigation of GCSCs holds significant potential in the eradication of solid tumors, particularly GC. However, the isolation and characterization of GCSCs have consistently posed a challenging obstacle that necessitates solutions for GCSCs research 16. Therefore, finding specific and effective cell surface markers for GCSCs is crucial for isolating and characterizing GCSCs. Increasing evidence confirms the presence of some specific cell surface markers in GCSCs 17. Studies have shown that CD44, CD133, CD90, and OCT4 can be used as GCSCs surface markers 18-23, which are associated with the properties of GCSCs, such as self-renewal and the ability to promote tumor progression. In addition to the isolation of GCSCs by cell surface markers, some features of GCSCs have been applied to isolate and identify GCSCs. Some scholars employed a serum-free media in the cultivation of GC cells AGS and HGC-27 to enrich GCSCs 24 To identify these cells, certain stemness markers were utilized, including Nanog, OCT4, and CD133. The introduction of growth factors into a serum-free suspension medium facilitates the formation of spherical cells and sustains the self-renewal capabilities of GCSCs. In contrast, non-GCSCs are unable to survive under these conditions. Consequently, this method is extensively employed to procure tumor cells exhibiting stem cell characteristics 25. The serum-free suspension culture technique is considered to be the simplest way to obtain GCSCs 26. Our group successfully enriched GCSCs using the serum-free suspension culture technique 27. The GCSCs were obtained from the GC cells SGC-7901 by serum-free suspension culture technique, and OCT4 and CD44 as identification markers for GCSCs. The findings from the experiment demonstrated that the cells cultivated in a serum-free suspension exhibited a spherical morphology. Additionally, the outcomes obtained from WB and RT-qPCR analyses revealed a significant upregulation in the expression of stemness markers CD44 and OCT4 in the enriched GCSCs. These results provide strong evidence that the experimental procedure successfully enriched GCSCs possessing stem cell characteristics.

In recent times, there has been a notable rise in the incidence of malignant tumors, leading to an increasing focus on anti-tumor therapy within the medical community. AQP5, a significant constituent of water channel proteins, has high expression levels within several tumor tissues and plays a crucial role in the progression of tumorigenesis 28, 29. The knowledge of the structural and functional characteristics of AQP5 has the potential to yield novel therapeutic interventions for neoplastic conditions. Previous studies have provided evidence supporting the utilization of AQP5 as a reliable indicator for the identification and enrichment of normal gastric pyloric stem cells 30. It has been observed that AQP5^+^ cells serve as the origin of invasive GC. Additionally, AQP5 expression is frequently detected in primary intestinal/diffuse GC as well as in its metastatic foci. The results of our WB and RT-qPCR tests indicated a considerable upregulation of AQP5 expression in enriched GCSCs. This finding suggests a potential involvement of AQP5 in the functioning of GCSCs. To conduct a more comprehensive examination of the impact of AQP5 on GCSCs, the present study established a stable model with low expression of sh-AQP5. The results revealed that the suppression of AQP5 hindered the proliferation, migration, and sphere-forming capacity of GCSCs. Conversely, the overexpression of AQP5 enhanced the self-renewal capability of GCSCs. These findings suggest that AQP5 may serve as a novel indicator for GCSCs and exert influence on the malignant biological activities of GCSCs.

We found that inhibition of AQP5 expression suppressed autophagy of GCSCs, which was reversed by overexpression of AQP5, and the autophagy inhibitor CQ reversed the spheroid-forming ability of GCSCs induced by AQP5, suggesting that AQP5 can affect the self-renewal ability of GCSCs through autophagy. In exploring the mechanism, we found that AQP5 interacts directly with TRPV4 by querying the STRING protein interactions prediction website, but some studies have also suggested that AQP5 acts synergistically with TRPV4 31. Our study showed that overexpression of AQP5 in GC cells was able to upregulate TRPV4 expression, and knockdown of AQP5 resulted in decreased TRPV4 expression, suggesting that there is a regulatory effect of AQP5 on TRPV4. TRPV4 is a classical Ca^2+^ channel, and TRPV4 is stimulated to activate it, and the rapid increase of intracellular Ca^2+^ can regulate the downstream signaling pathways, which in turn affects the different processes of tumorigenesis. Numerous studies have confirmed that Ca^2+^ intracellular flow can activate cellular autophagy 32, and our experimental results demonstrated that knocking down the expression of TRPV4 inhibited the expression of the autophagy protein LC3 and the GC stemness marker gene OCT4 in GCSCs, and that knocking down TRPV4 was able to reverse autophagy and self-renewal capacity of GCSCs induced by overexpression of AQP5. This suggests that AQP5 can regulate TRPV4 to affect the autophagy and self-renewal capacity of GCSCs. However, additional investigation and refinement are required to elucidate the precise molecular mechanism involved.

## Conclusion

In conclusion, the present study revealed a significant upregulation of AQP5 expression in GCSCs. Moreover, it was shown that AQP5 potentially influences the progression of GC through its regulatory role in autophagy. These findings offer novel diagnostic insights and therapeutic prospects for the management of GC.

It can be concluded that AQP5 is highly expressed in GCSCs, and it can affect the self-renewal ability of GCSCs by activating autophagy. AQP5 may be one of the important targets for GC therapy.

## Figures and Tables

**Table 1:** Correlation between the expression of AQP5 and clinicopathologic markers in GC tissues

	N (N=73)	AQP5		χ^2^	P value
		High, n=46	Low, n=27		
Gender					
Male	55	37	18	1.736	0.188
Female	18	9	9		
Age (years)					
< 65	42	27	15	0.069	0.793
≥ 65	31	19	12		
Degree of differentiation					
Well/moderate	33	22	11	0.345	0.557
Poor	40	24	16		
Tumor size (cm)					
< 4cm	29	14	15	4.484	0.034*
≥ 4cm	44	32	12		
TNM stage					
I	16	8	8	2.338	0.311
II	25	15	10		
III	32	23	9		
Depth of tumor invasion					
T1	16	9	7	1.485	0.686
T2	6	5	1		
T3	31	20	11		
T4	20	12	8		
Lesion site					
Upper	25	15	10	0.202	0.904
Middle	18	12	6		
Lower	30	19	11		
Lymph node metastasis					
No	26	11	15	7.429	0.006**
Yes	47	35	12		
Vascular invasion					
No	64	39	25	0.960	0.327
Yes	9	7	2		

**Notes:** *P < 0.05, **P < 0.01.

**Table 2:** Univariate and Multivariate Cox Regression Analyses of Overall Survival in Patients with GC

Multivariate analysis	Univariate analysis		Multivariate analysis	
	Log-rank χ^2^	P value	HR (95% CI)	P value
Gender				
Male vs female	2.44	0.118		
Age (years)				
< 65 vs ≥ 65	0.091	0.763		
Degree of differentiation				
Well/moderate vs poor	3.660	0.056		
Tumor size (cm)				
< 4 vs ≥ 4	3.664	0.056		
TNM Stage				
I+II vs III	9.880	0.002**	1.282 (0.533-3.084)	0.579
Depth of tumor invasion				
T1-T2 vs T3-T4	6.703	0.010**	2.142 (0.804-5.707)	0.128
Lesion site				
Upper and middle vs lower	0.031	0.860		
Lymph node metastasis				
Not vs Yes	7.139	0.008**	0.859 (0.341-2.163)	0.747
Vascular invasion				
Not vs Yes	4.818	0.028*	0.277 (0.115-0.668)	0.004**
AQP5 expression				
Low vs high	14.416	< 0.001***	4.856 (2.050-11.504)	< 0.001***

**Notes:** *P < 0.05, **P < 0.01, ***P < 0.001.

**Table 3:** Correlation Between Expression of AQP5 and CD133 in GC

	AQP5 expression		r	P value
	High, n	Low, n		
CD133 expression				
Low, n	20	16	0.311	0.007**
High, n	26	11		

**Notes:** **P < 0.01.
